# Preparation, Structural Characterization, and Stability of Low-Molecular-Weight Collagen Peptides–Calcium Chelate Derived from Tuna Bones

**DOI:** 10.3390/foods12183403

**Published:** 2023-09-12

**Authors:** Yaqi Zhong, Yufang Zhou, Mingzhu Ma, Yadong Zhao, Xingwei Xiang, Conghan Shu, Bin Zheng

**Affiliations:** 1 School of Food and Pharmacy, Zhejiang Ocean University, Zhoushan 316000, China; zhongyq1997@163.com (Y.Z.); yadong@kth.se (Y.Z.); shuconghan@163.com (C.S.); 2 Zhejiang Marine Development Research Institute, Zhoushan 316000, China; 18363010902@163.com; 3 Science and Technology Development Center, Zhejiang Marine Development Research Institute, Zhoushan 316000, China; 4 College of Food Science and Technology, Zhejiang University of Technology, Hangzhou 310014, China

**Keywords:** tuna bone collagen peptides, peptides–calcium chelate, structural characterization, morphological properties, stability

## Abstract

This study was conducted to prepare calcium chelate of low-molecular-weight tuna bone collagen peptides (TBCP_LMW_) with a high chelation rate and to identify its structural characteristics and stability. The optimum conditions for calcium chelation of TBCP_LMW_ (TBCP_LMW_-Ca) were determined through single-factor experiments and response surface methodology, and the calcium-chelating capacity reached over 90% under the optimal conditions. The amino acid compositions implied that Asp and Glu played important roles in the formation of TBCP_LMW_-Ca. Structural characterizations determined via spectroscopic analyses revealed that functional groups such as -COO^−^, N-H, C=O, and C-O were involved in forming TBCP_LMW_-Ca. The particle size distributions and scanning electron microscopy results revealed that folding and aggregation of peptides were found in the chelate. Stability studies showed that TBCP_LMW_-Ca was relatively stable under thermal processing and more pronounced changes have been observed in simulated gastric digestion, presumably the acidic environment was the main factor causing the dissociation of the TBCP_LMW_-Ca. The results of this study provide a scientific basis for the preparation of a novel calcium supplement and is beneficial for comprehensive utilization of tuna bones.

## 1. Introduction

Calcium is a vital nutrient for human health, accounting for about 1.5–2% of human body weight [1]. Calcium deficiency may provoke a series of diseases, such as osteopenia, osteoporosis, high blood pressure, and kidney stones [2]. Currently, there are a variety of commercial calcium supplements, including inorganic calcium, organic calcium [3,4]. Nevertheless, the poor water solubility and relatively low absorption rate of calcium lead to its low bioavailability in vivo [5,6,7]. Amino acid chelates have been reported to have a higher absorption rate [8], but the use of high-purity amino acids is quite expensive and may produce uncontrollable colour generation and oxidation reactions [9,10]. Studies have shown that food-derived peptides chelated with minerals have high stability, absorption, and bioavailability [9,11]. Calcium combined with peptides could form a complex with the advantages of fast absorption, less saturation, and lower energy consumption compared to amino acid calcium [12]. Therefore, peptides–calcium chelates have promising potential as functional food additives [13].

In recent years, calcium chelates of food-derived peptides have attracted significant attentions owing to their high stability, absorption rate, and bioavailability [5]. Various calcium-binding peptides have been prepared from aquatic organism. Wu et al. developed novel octopus scraps peptides with calcium-chelating activity [14]. Cui et al. identified the calcium-binding peptides from sea cucumber eggs and explored their calcium-binding modes [15]. Some laboratories have conducted preparation technology studies as well as structural and functional studies on calcium chelation with fish collagen peptides from Pacific cod bone [16], European eel bone [17], and tilapia bone [18]. Furthermore, it has also been found that lower-molecular-weight peptides have better chelating effects [19,20]. Tuna is a highly commercial marine fish that contributes significantly to the development of world fisheries. The processing of canned tuna generates plenty of by-products, including bones, viscera, red meat, and so on, which are usually processed into fishmeal or discarded as waste [21]. In particular, tuna bones account for a large proportion of the by-products, and they are rich in collagen and calcium, but their utilization is extremely inadequate [22,23]. Previous studies have demonstrated that collagen peptides have antioxidant, osteogenic, immunomodulatory, and anti-inflammatory activities [5,7,18]. Therefore, it is critical to utilize the abundant tuna bone resources to produce valuable collagen peptides-related products.

In this research, peptides–calcium chelates were prepared from low-molecular-weight tuna bone collagen peptides (TBCP_LMW_). Chelating conditions of TBCP_LMW_ and calcium ions were optimized. Structural characterizations and morphological analysis were also investigated. The stability of the peptides–calcium chelate was assayed under different conditions and through simulated gastrointestinal digestion in vitro. The findings of this study would provide a scientific basis for the application of tuna bone collagen peptides–calcium chelate (TBCP_LMW_-Ca) and would improve the utilization of tuna bones.

## 2. Materials and Methods

### 2.1. Materials and Chemicals

Tuna bones were obtained from Zhejiang Retronx Foodstuff Industry Co., Ltd. (Zhoushan, China) and stored at −20 °C before use. Pepsin (EC 3.4.23.1, ≥2000 U/g) was purchased from Chinachem Shuanghui Industrial Company Biochemical Pharmaceutical Factory (Tahe, China). Animal protease (≥100,000 U/g) was purchased from Nanning Doing-higher Bio-tech Co., Ltd. (Nanning, China). Acetonitrile (≥99.9%) and trifluoroacetic acid (≥99.5%) were purchased from Macklin (Shanghai, China). Peptide standards were purchased from WEIYE Metrology and Technology Research Group Co. (Beijing, China). All other reagents in the experiment were analytically pure.

### 2.2. Preparation of TBCP_LMW_

TBCP_LMW_ was extracted according to the methods developed by our laboratory previously. Details were as follows: Tuna bones were cleaned to remove any remaining flesh and sinew under water, and then they were cut into 2–3 cm pieces with scissors. The cleaned bones were mixed with distilled water (1:10, *w*/*v*) and treated with pepsin (2%) at 55 °C for 3 h (pH 3.0). Pepsin was subsequently inactivated at 100 °C for 15 min. The enzymatically digested bones were steamed under 121 °C, 0.12 MPa for 30 min to remove the fat and reduce the hardness and toughness of tuna bones. After filtering the residue, the cleaned bones were placed in 2% edible sodium bicarbonate solution for 120 min and then dried and smashed into powders. The tuna bone powders were added to distilled water at a ratio of 1:10 (*w*/*v*), enzymatically digested by adding animal protease (2%, pH 7.0) at 50 °C for 8 h, and then inactivated at 100 °C for 15 min. The supernatant was collected after centrifugation at 11,800× *g* (TG16-WS, Cenen, Changsha, China) for 15 min (4 °C) and passed through the spiral-wound membranes with cutoff of 1000 Da and 250 Da, respectively, by using multi-functional rolled membrane pilot equipment (RNF-0460, Starmem, Xiamen, China). Then, the fractions in 250–1000 Da solution were collected and lyophilized.

### 2.3. Preparation of TBCP_LMW_-Ca

Single-factor experiments were conducted, taking the calcium-chelating capacity as a screening index. The prepared TBCP_LMW_ was dissolved in 10 mL ultrapure water, and then it was mixed with different mass ratios of CaCl_2_ (1:2, 1:1, 2:1, 3:1, 4:1) at 30 °C, 50 °C, 70 °C, and 90 °C for 1 h, 2 h, 4 h, and 6 h. pH was adjusted to 5.0, 6.0, 7.0, 8.0, and 9.0. After chelation, anhydrous ethanol (9-fold volume) was added to separate the chelate. The mixture was then centrifuged at 7552× *g* (TG16-WS, Cenen, Changsha, China) for 10 min, and the precipitate was collected and freeze-dried to obtain TBCP_LMW_-Ca. A three-level, four-factor Box–Behnken Design was carried out to determine the optimal conditions based on the one-way test using four independent variables: peptide/calcium mass ratio (A), pH (B), chelation time (C), and chelation temperature (D) (Table 1). The total amount of calcium in the chelating solution and the calcium content in the supernatant after centrifugation was both determined via the EDTA titration method [24].

The calcium-chelating capacity was calculated as follows:

$$\mathrm{Chelation}\;\mathrm{rate}\;(\%)=\frac{Total\;calcium\;ions\;content/mg\;-\;Free\;calcium\;ion\;content/mg}{\begin{array}{c} \mathrm{Total}\;\mathrm{calcium}\;\mathrm{ions}\;\mathrm{content}/\mathrm{mg} \end{array}}\;\times \;100$$

### 2.4. Amino Acid Composition Analysis

The TBCP_LMW_ and TBCP_LMW_-Ca were transferred into the hydrolysis tube, respectively, and 15 mL of hydrochloric acid solution (6 mol/L) was added to mix the samples thoroughly, followed by 3–4 drops of phenol for hydrolysis. The tubes were pumped close to 0 Pa, filled with nitrogen 3 times/min to remove the air and then sealed while full of nitrogen. The sealed hydrolysis tubes were transferred to an electric blast thermostat at 110 °C, 22 h. After being filtered through a 0.22 μm filter, the solutions were analysed using an automatic amino acid analyser (L8900, Hitachi, Tokyo, Japan).

### 2.5. Molecular Weight Distribution

The molecular weight distribution was analysed using an Agilent 1260 HPLC (Santa Clara, CA, USA). Chromatographic column was TSKgel 2500 SWXL (300 mm × 7.8 mm, TOSOH, Tokyo, Japan); mobile phase: acetonitrile/water/trifluoroacetic acid, 45/55/0.1 (*v*/*v*); injection volume 10 μL; column temperature 25 °C; flow rate 0.5 mL/min; detection wavelength 220 nm. Aprotinin (6511 Da), Bacitracin (1422 Da), Gly-Gly-Tyr-Arg (451 Da), and Gly-Gly-Gly (189 Da) were used as standards and configured to 1 mg/mL using the mobile phase. TBCP_LMW_ and TBCP_LMW_-Ca were also diluted to 1 mg/mL using the mobile phase, filtered through a 0.45 μm filter, and then subjected to subsequent experiments.

### 2.6. Structural Characterization

#### 2.6.1. Fluorescence Spectroscopy Determination

TBCP_LMW_ and TBCP_LMW_-Ca were configured to 1 mg/mL using deionized water. The fluorescence spectra were measured using a fluorescence spectrophotometer (Cary3500, Agilent, Santa Clara, CA, USA). The excitation wavelength was fixed at 295 nm. The emission wavelength was ranged from 280 to 500 nm.

#### 2.6.2. Ultraviolet Spectroscopic Determination

TBCP_LMW_ and TBCP_LMW_-Ca were prepared into an aqueous solution at a mass concentration of 1 mg/mL. Change in UV absorption wavelength was measured by UV spectrophotometer (Cary60, Agilent, Santa Clara, CA, USA) at the scanning wavelength of 190–400 nm.

#### 2.6.3. Fourier-Transform Infrared (FTIR) Spectroscopy Analysis

The experiments were performed in dry air at (25 ± 1) °C. A 1 mg sample was firstly mixed with KBr and then pressed into a thin transparent sheet. FTIR spectra of TBCP_LMW_ and TBCP_LMW_-Ca were observed using an FTIR spectrometer (TensorII, Bruker, Saarbrucken, Germany) at 400–4000 cm^−1^, and 64 scans of each spectrum were collected.

#### 2.6.4. Circular Dichroism (CD) Spectroscopy

TBCP_LMW_ and TBCP_LMW_-Ca solutions were prepared to 0.2 mg/mL. Spectra were scanned using circular dichroism spectrometer (MOS-500, Bio-Logic, Seyssinet-Pariset, France) in the range of 185–260 nm, at a scanning rate of 100 nm/min and an interval of 1 nm.

### 2.7. Particle Size Distribution and Zeta Potential Analysis

The samples were dissolved in ultrapure water and configured to 1 mg/mL. The size distribution and ζ-potential were figured by Zetasizer Nano instrument (ZS90, Malvern Instruments Ltd., Malvern, UK) at 25 °C.

### 2.8. Scanning Electron Microscopy

The microscopic morphology of TBCP_LMW_ and TBCP_LMW_-Ca was observed using a scanning electron microscope (Sigma 300, ZEISS, Oberkochen, Germany). Sample powders were placed on the conductive adhesive holder and sprayed with gold at 10 mA, and the samples were observed at 8000× magnification under an accelerating voltage of 2.0 kV.

### 2.9. Stability Analysis of TBCP_LMW_-Ca

#### 2.9.1. Acid–Base Stability

The lyophilized powder of TBCP_LMW_-Ca was dissolved in deionized water to 10 mg/mL in each tube. pH was 3, 4, 5, 6, 7, 8, and 9, respectively. The reaction was carried out in a water bath at 37 °C for 1 h. After the reaction, calcium ion content was analysed via the colourimetric method using o-phenolphthalein [14]. The acid–base stability of TBCP_LMW_-Ca was calculated as calcium retention rate of the chelate after the reaction.

#### 2.9.2. Thermal Stability

The lyophilised powder of TBCP_LMW_-Ca was dissolved in deionised water (pH 7). The final concentration of each tube was 10 mg/mL. The reaction was conducted for 1 h at 50 °C, 60 °C, 70 °C, 80 °C, 90 °C, and 100 °C, respectively. The thermal stability of TBCP_LMW_-Ca was calculated as calcium retention rate of the chelate after the reaction.

#### 2.9.3. Simulated Digestion In Vitro

A standard static in vitro digestion model (INFOGEST) [25] was applied with minor modifications to assess the stability of TBCP_LMW_-Ca in simulated gastrointestinal digestion. Stability was calculated as the retention of calcium in TBCP_LMW_-Ca after digestion. In the gastric phase, TBCP_LMW_-Ca was dissolved using the simulated gastric fluid electrolyte in a 1:1 ratio. Porcine pepsin was added to achieve an activity of 2000 U/mL. At the same time, pre-prepared gastric lipase solution was added to achieve 60 U/mL gastric lipase activity in the mixture. After regulating the pH to 3, the TBCP_LMW_-Ca concentration was adjusted to 10 mg/mL. In the intestinal stages, TBCP_LMW_-Ca was dissolved in a 1:1 ratio using a simulated intestinal fluid electrolyte and added to the hepatic bile water compound for 30 min. Meanwhile, trypsin, pancreatic rennet, pancreatic α-amylase and pancreatic lipase were added to reach 100, 25, 200, and 2000 U/mL, respectively. pH was adjusted to 7, and the reaction was timed from this point onwards. In the gastrointestinal phase, all previous steps were repeated, with a simulated gastric reaction for 90 min, followed immediately by adjustment of pH to add the enzyme required for the intestinal phase. Each stage of the reaction was carried out at 37 °C in a constant temperature water bath shaker. The enzyme was then inactivated at 100 °C for 15 min. The content of calcium ions in the solution was measured every 30 min using the colourimetric method with o-phenolphthalein. Stability of TBCP_LMW_-Ca in simulated gastrointestinal digestion was calculated and expressed as calcium retention rate after digestion.

### 2.10. Statistical Analysis

All measurements were performed thrice in parallel, and all data were presented as mean ± standard deviation. SPSS 26 was used for data analysis. Statistical significance was determined via Duncan’s multiple range test of one-way ANOVA analysis. *p* < 0.05 was considered as statistically significant.

## 3. Results and Discussion

### 3.1. Preparation of TBCP_LMW_-Ca

#### 3.1.1. Single Factor Experiments

The calcium chelates of collagen peptides extracted from various sources have been reported as potential calcium supplements, such as cod [26], tilapia [27], sheep bone, and bovine bone [28,29], and the processing conditions could affect the calcium-chelating capacity of the peptides.

**Figure 1:**
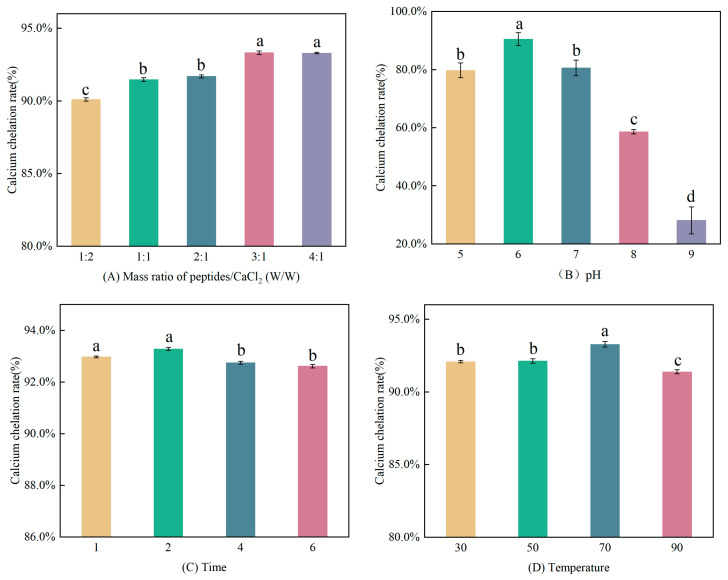
Effects of four factors on TBCP_LMW_ calcium-chelating capacity. (**A**) Peptide/calcium mass ratio, (**B**) pH, (**C**) Time, and (**D**) Temperature. a,b,c,d: statistical significance.

In the study, the effects of different factors on calcium chelation rate were investigated. As depicted in Figure 1A, when the peptide/calcium mass ratio increased gradually, the capacity to chelate calcium increased (*p* < 0.05), and there was no significant difference in calcium-chelating ability when the mass ratio exceeded 3:1. According to Figure 1B, when the pH value varied between 5 and 8, the calcium-chelating capacity firstly increased from 5 to 6 (*p* < 0.05), peaked at pH 6 with a chelating capacity of 90.47%, and decreased gradually when the pH exceeded 6 (*p* < 0.05). It implied that pH value effected the calcium chelation rate significantly, consistent with the conclusion of Luo et al. [5]. The change might be owing to the fact that as the pH value increased, OH^−^ competed with the electron donor group for Ca^2+^, preferentially binding to produce calcium hydroxide precipitates, which was not conducive to chelate. As the pH value decreased, the interaction between Ca^2+^ and peptides would be destroyed by the large amount of H^+^ [5]. As shown in Figure 1C, the chelation reaction proceeded rapidly in a short period of time. The calcium-chelating capacity was higher when the reaction time was 1 h or 2 h than that of 4 h and 6 h (*p* < 0.05). It can be speculated that the prolonged reaction time may have led to some decomposition of TBCP_LMW_-Ca and thus reduced the calcium-chelating capacity. According to Figure 1D, when the temperature arose from 30 °C to 70 °C, the calcium-chelating capacity increased, and it reached the maximum at 70 °C. The calcium-chelating capacity declined notably when the temperature rose to 90 °C. Chelation reaction is a dynamic equilibrium process. An appropriate temperature will accelerate the molecular motion and improve the chelation rate, while an excessive temperature would change the conformation of peptides and hinder chelation [29,30].

#### 3.1.2. Response Surface Optimization

Experiments were designed with the peptide/calcium mass ratio (A), pH (B), chelation time (C), and chelation temperature (D) as response variables, with calcium-chelating capacity as the response value, with three levels for each variable and equidistance between each level. A total of 29 sets of experiments were conducted, including five sets of centre point replicates, and the results are displayed in Table 2.

The results of the RSM experiments were analysed. Multiple regression was fitted to give the following quadratic multinomial regression model equation: Y = 94.08 + 1.01A + 2.02B + 0.19C − 0.7D + 0.085AB − 0.12AC − 0.017AD + 0.11BC + 0.26BD − 0.11CD − 0.86A^2^ − 7.51B^2^ − 1.04C^2^ − 2.57D^2^. The model was subjected to an ANOVA, and the results are depicted in Table 3. *p*-value of the regression equation model was lower than 0.01, indicating a good fit of the equation. The lack-of-fit value of 0.1110 was not significant relative to the pure error, which indicated that the model was a reasonable choice. The correlation coefficient (R^2^ = 0.9908) was relatively reliable. Therefore, this model could be used to analyse and predict the chelation of TBCP_LMW_ with calcium chloride. Among the variables, peptide/calcium mass ratio, pH, and temperature all had extremely significant effects on the chelation rate (*p* < 0.01), while the effect of chelation time was not significant (*p* > 0.05). The squared terms of all factors were extremely significant (*p* < 0.01).

According to the model, the following optimum chelating conditions were recommended: peptides/calcium mass ratio 3.29:1, pH 6.14, time 2.04 h, temperature 68.69 °C, with a theoretical chelation rate of 94.56%. For practical considerations, the predicted process was modified: peptide/calcium mass ratio 3.3:1, pH 6.1, time 2 h, and temperature 69 °C. The maximum actual chelation rate measured under these conditions was 94.27%. The chelation rate of this optimum model was slightly higher than the findings of Wu et al. [31]. The use of TBCP_LMW_ to prepare calcium chelate under this optimum condition was shown to be advantageous and worthy of further study.

### 3.2. Amino Acid Composition

**Figure 2:**
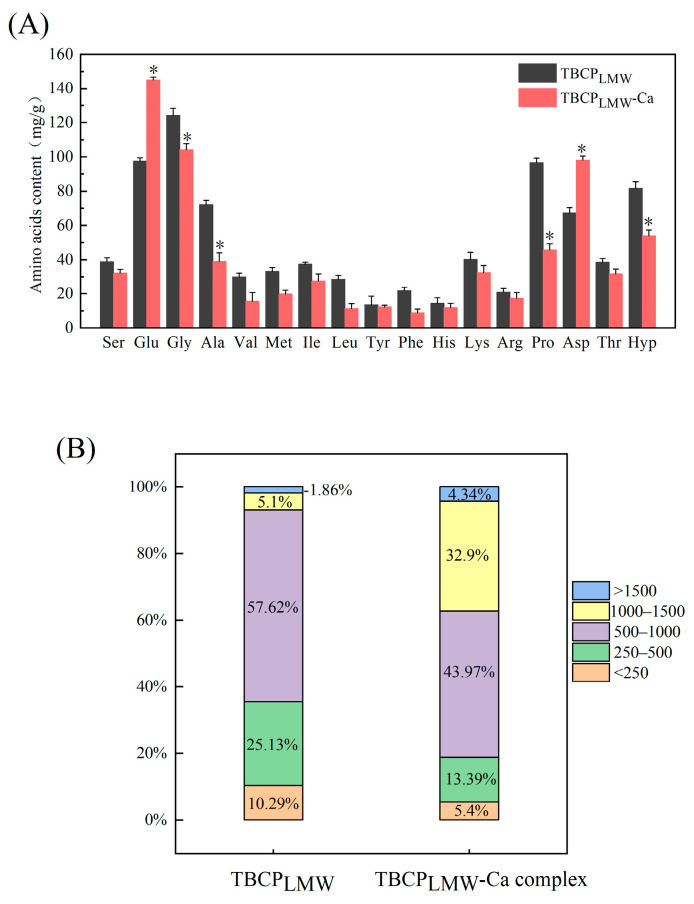
(**A**) Amino acid analysis of TBCP_LMW_ and TBCP_LMW_-Ca. (**B**) Molecular weight distribution of TBCP_LMW_ and TBCP_LMW_-Ca complex. The asterisk symbols indicate statistical significance (*p* < 0.05).

Amino acid compositions of TBCP_LMW_ and TBCP_LMW_-Ca were presented in Figure 2A. They were abundant in Glu, Asp, Gly, Hyp, Pro, etc., which confirmed the characteristics of collagen peptides, and frequently present in the mineral-binding peptides [5,32]. Compared to TBCP_LMW_, TBCP_LMW_-Ca showed a significant increase in the contents of Asp and Glu. According to previous studies, acidic amino acids have been recognized as essential amino acids, and they may facilitate the calcium-chelating capacity of proteins and peptides, owing to the free carboxyl groups [24,33]. It was in line with the research of Zhang et al. that Asp was one of the important components involved in the calcium chelation of the decapeptide from Pacific cod bone hydrolysate, and Glu formed peptide calcium chelates by binding to calcium ions through multiple carboxylic acid groups [16]. All the results indicated that Asp and Glu played important roles in the formation of TBCP_LMW_-Ca.

### 3.3. Molecular Weight Distribution

Molecular weight distribution of TBCP_LMW_ and TBCP_LMW_-Ca were presented in Figure 2B, and the chromatograms are displayed in Figure S1. TBCP_LMW_ was mainly concentrated below 1000 Da, with 250–1000 Da accounting for 82.75%. It has been reported that fish collagen peptides between 180 and 2000 Da have better calcium-chelating ability [19], and peptides with smaller molecular weights are more favourable for chelating calcium ions [34]. Therefore, in this study, low-molecular-weight peptides were selected for the preparation of peptide calcium chelate.

Although a certain calcium dissociation of TBCP_LMW_-Ca existed in the mobile phase due to the acidic environment, the molecular weight distribution of TBCP_LMW_-Ca complex differed from TBCP_LMW_-Ca. As depicted in Figure 2B, the percentage of 1000–1500 Da was 32.9% after chelation, while that of TBCP_LMW_ was 5.1%. The main reason may be the formation of new chelation bonds, which increased the peptide crosslinks. Similar changes in molecular weight were reported during the preparation of calcium chelates from bovine collagen peptides [35].

### 3.4. Structural Characterization

#### 3.4.1. Fluorescence Spectroscopy

**Figure 3:**
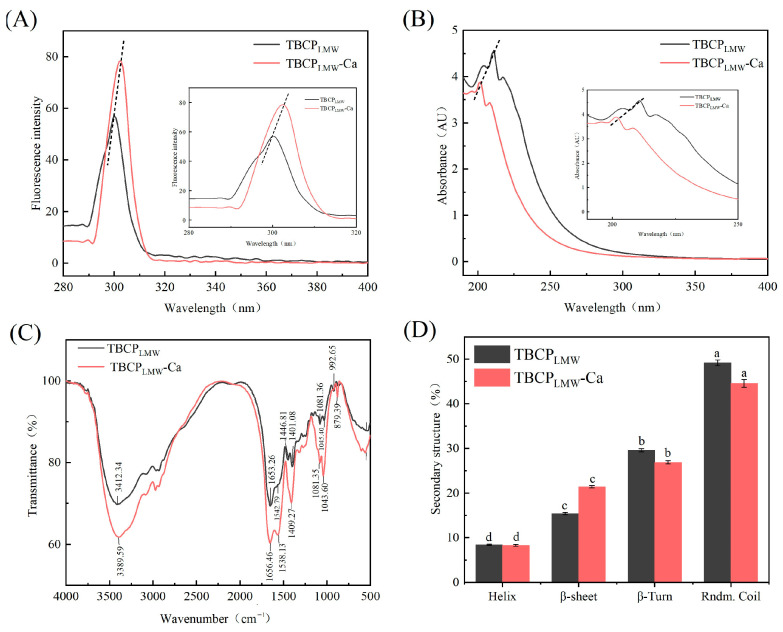
Structural characterization of TBCP_LMW_ and TBCP_LMW_-Ca: (**A**) Fluorescence spectroscopy, (**B**) UV absorption spectroscopy, (**C**) FTIR analysis, and (**D**) CD spectroscopy. a,b,c,d: statistical significance.

Structural changes between amino acid groups and mineral ions could be judged by the wavelength and intensity changes in fluorescence spectroscopy [36]. The fluorescence results in Figure 3A showed that TBCP_LMW_-Ca showed a red shift and an increase in endogenous fluorescence intensity at the excitation wavelength of 295 nm. This enhancement may be due to the complex interaction between the chromophore and calcium leading to a change in excited state energy, which in turn affected the intensity. This is consistent with the results of previous studies [24,37], which showed that the chelation of calcium by TBCP_LMW_ led to structural changes in amino acids and peptides, resulting in folding and aggregation, and indicated the successful binding of TBCP_LMW_ to calcium ions.

#### 3.4.2. UV–Vis Absorption Spectroscopy Assay

Changes in intensity and dislocation in the UV absorption spectra can reflect the differences between peptides and calcium chelate [38]. According to Figure 3B, the absorption spectra of TBCP_LMW_ and TBCP_LMW_-Ca had multiple absorption peaks in the range of 190–280 nm. A higher absorption peak of TBCP_LMW_ occurred at 220 nm, which was consistent with the findings that the n→π* leap of C=O in the peptide bond generally occurs around 210 nm [39]. After chelating calcium, it can be seen from the chromatogram that the UV absorption spectrum was shifted considerably, and new substances were produced. Moreover, the absorption intensity of the closing peak of TBCP_LMW_-Ca at 201 nm was reduced, and a colour reduction effect was observed, which may be the result of the chelation of carboxyl and amino groups with calcium ions [40]. The results of the spectra demonstrated that TBCP_LMW_ reacted with calcium ions, and TBCP_LMW_ in the near-UV region represented higher absorbance than that of TBCP_LMW_-Ca, which may be due to the spatial structure changes in TBCP_LMW_ caused by binding with calcium ions.

#### 3.4.3. Fourier-Transform Infrared Spectroscopy

FTIR spectrum is a good reflection of the differences between two substances, and the changes in the characteristic absorption peaks reflect the interaction of the mineral ions with the organic groups of the protein [41].

According to Figure 3C, the peak of TBCP_LMW_ at 3412.34 cm^−1^ shifted to 3389.59 cm^−1^ after calcium chelation, corresponding to the stretching vibration of N-H, indicating that N-H contributed to the formation of the chelate. The amide I band of TBCP_LMW_ shifted from 1653.26 cm^−1^ to 1656.46 cm^−1^, relating to the stretching vibration of the carbonyl group in the peptide bonds, which demonstrated that C=O was involved in the formation of TBCP_LMW_-Ca. In addition, the amide II peak of TBCP_LMW_ moved from 1542.79 cm^−1^ to 1538.13 cm^−1^ in TBCP_LMW_-Ca, representing the bending vibration of N-H, implying that -NH_2_ might react with calcium. The absorption wave number 1401.08 cm^−1^ moved to a higher frequency of 1409 cm^−1^ after chelation, while the vibrational spectral region of 1430–1370 cm^−1^ was associated with the stretching vibrations of carboxylate group. It suggested that -COO^−^ may be bound to calcium and converted to -COOCa, which was consistent with previous findings [36]. The 1300–600 cm^−1^ region represents the fingerprint region of the compound. After the chelation occurred, the peak at 1081.36 cm^−1^ moved to 1043.60 cm^−1^, indicating that the C-O bond might participate in the chelation and formed new coordination bonds with calcium [42]. Therefore, it can be speculated that -COO^−^, N-H, C=O, and C-O groups might be involved in the formation of TBCP_LMW_-Ca.

#### 3.4.4. Circular Dichroism

The protein secondary structure can be analysed by circular dichroism spectra [43]. As shown in Figure 3D, the β-sheet in TBCP_LMW_-Ca increased from 15.4% to 21.4%, which led to the conclusion that calcium ions could promote β-sheet production [24,34]. The binding of calcium to TBCP_LMW_ decreased both α-helix and random folding. It is hypothesized that the binding of calcium may increase the exposure of hydrophobic groups and lead to the decay of intramolecular hydrogen bonds, thus reducing the α-helix as well as increasing the β-sheet. The reduction in random folding suggested that TBCP formed a tighter secondary structure upon binding to calcium ions, similarly to that of previous research by Zhang et al. [16] and Yang et al. [44].

### 3.5. Zeta Potential and Particle Size Distribution

**Figure 4:**
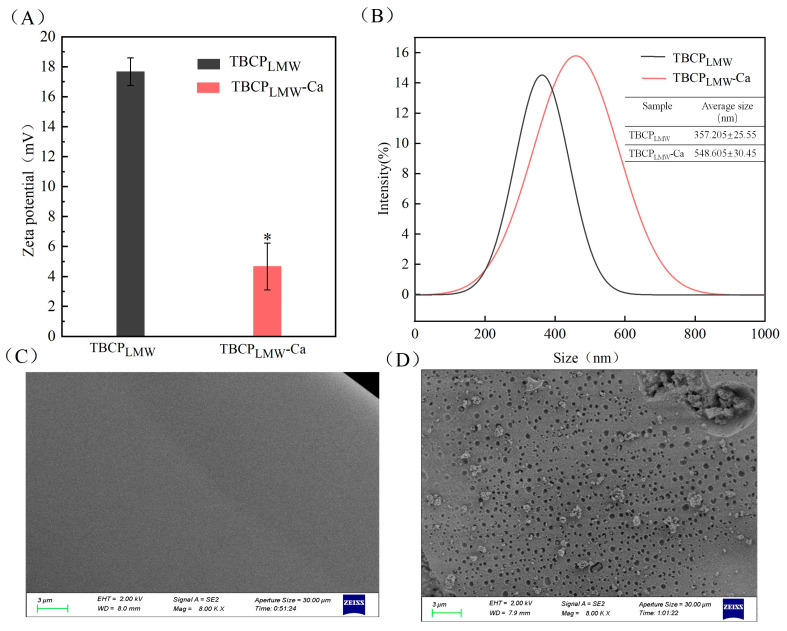
(**A**) Zeta potential, (**B**) Particle size distribution, (**C**) SEM image of TBCP_LMW_ at 3 μm, and (**D**) SEM image of TBCP_LMW_-Ca at 3 μm. The asterisk symbols indicate statistical significance (*p* < 0.05).

The surface charge of protein particles was assayed through Zeta potential measurement [45]. As depicted in Figure 4A, the value of TBCP_LMW_-Ca was significantly lower compared to TBCP_LMW_, dropping from 17.67 mv to 4.66 mv. It suggested that a transfer of electrons between TBCP_LMW_ occurred when they reacted with calcium. It could be the negative charge of TBCP_LMW_ was neutralized by calcium ions [46].

Particle size distributions of TBCP_LMW_ and TBCP_LMW_-Ca are displayed in Figure 4B. The mean particle size of TBCP_LMW_-Ca increased from 285.13 nm to 313.45 nm. An increase in the mean particle size was observed after chelation, presumably due to the mineral ions acting as a salt bridge by shielding the negative charge on the peptide chain and promoting protein aggregation [37].

### 3.6. Scanning Electron Microscope

SEM images of TBCP_LMW_ and TBCP_LMW_-Ca in magnification of 8000 are displayed in Figure 4C,D. The surface of TBCP_LMW_ was dense and smooth, while that of TBCP_LMW_-Ca was rough with a large number of pores and granular aggregates. The differences between TBCP_LMW_ and TBCP_LMW_-Ca lied in the fact that the calcium chelation had destructed the dense structure of TBCP_LMW_. The amino and carboxyl groups of TBCP_LMW_ were combined with calcium ions, inducing protein aggregation. The results were consistent with the electron micrographs of bovine collagen peptide calcium chelate [28,35,44].

### 3.7. Stability

#### 3.7.1. pH and Temperature

**Figure 5:**
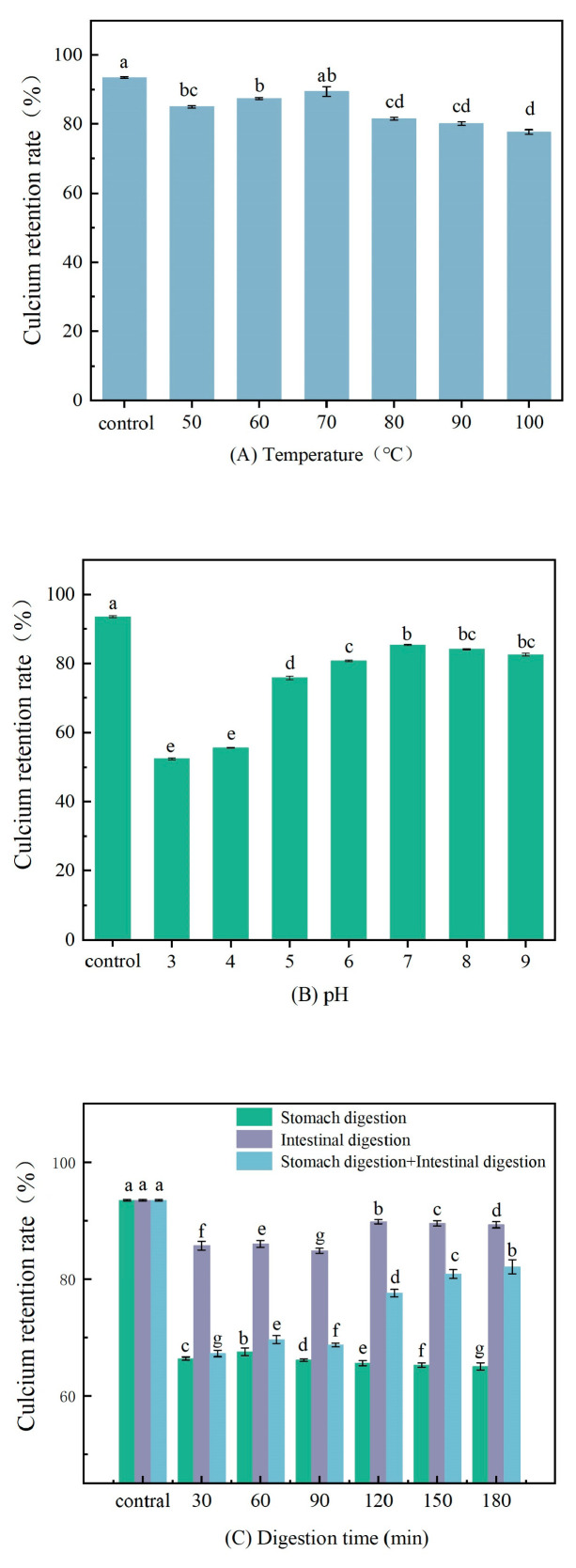
Stability of TBCP_LMW_-Ca: (**A**) at different pH, (**B**) at different temperatures, and (**C**) during simulated gastrointestinal digestion in vitro. a,b,c,d,e,f,g: statistical significance.

The calcium retention rate of TBCP_LMW_-Ca at different conditions of pH and temperatures are displayed in Figure 5A,B. The calcium retention decreased significantly at pH 3 and 4, indicating that high acidic conditions were not conducive to the retention of calcium in TBCP_LMW_-Ca. It was due to the fact that the abundant H^+^ may compete with Ca^2+^ and lead to dissociation of TBCP_LMW_-Ca [6]. A similar situation was found in porcine collagen peptide calcium chelate [31]. On the other hand, the calcium retention rate of TBCP_LMW_-Ca slightly decreased as the temperature changing from 50 °C to 90 °C, but it remained above 78%. It suggested that the chelate has great resistance to heating, similar to the peptide–calcium chelate from stickwater and oyster shells [47].

#### 3.7.2. In Vitro Digestion Simulation

Dietary nutrients usually pass through the stomach and reach the intestine, where they are digested, absorbed, and utilised. It has been shown that pepsin in the stomach can hydrolyse peptides into amino acids, resulting in adverse changes in the biological activity of the peptides [48]. The tolerance of TBCP_LMW_-Ca to gastrointestinal enzymes and its stability in the digestive environment was evaluated using the INFOGEST static digestion system.

As shown in Figure 5C, the reaction conditions chosen in the stomach alone were pH 3 with the addition of pepsin as well as gastric lipase, and the retention of calcium decreased to approximately 66.40% at 30 min intervals. This indicated that pH had a large effect on retention, while the addition of pepsin and gastric lipase may cause further hydrolysis of the peptide, so gastric environment may be the main stage for the release of calcium ions. In contrast, TBCP_LMW_-Ca had a higher stability in the intestinal alkaline digestive environment and was largely unaffected by bile as well as other digestive enzymes [31]. In the gastrointestinal phase, the calcium retention rate decreased and remained at about 67.28% for the first 90 min, while it increased significantly when converted to the intestinal environment. It implied that the intestinal environment can not only inhibit the dissociation of calcium ions from TBCP_LMW_-Ca, but also contribute to the re-chelation of TBCP_LMW_ and calcium [15].

It was clear from the results that the main factor leading to the dissociation of calcium ions from TBCP_LMW_-Ca was a change in pH, using an in vitro simulated digestion model combined with pH experiments. Pepsin, gastric lipase, pancreatic protease, and bile had little effect on TBCP_LMW_-Ca, and the weak alkaline environment in the intestine promoted re-chelation of calcium ions and peptides.

## 4. Conclusions

In this study, TBCP_LMW_-Ca were prepared to use low-molecular-weight collagen peptides obtained via enzymatic digestion combined with membrane grading technique from tuna bones, and the preparation conditions of TBCP_LMW_-Ca were optimized. The analysis of amino acid compositions showed that Asp and Glu may facilitate the chelation with calcium. The results of structural characterization revealed the changes in TBCP_LMW_ structure after calcium chelation. The molecular weight distribution and morphological analysis indicated that calcium ions cross-linked with collagen peptides, and aggregation occurred in TBCP_LMW_-Ca. The stability analysis investigated that TBCP_LMW_-Ca was comparatively stable at high temperatures and gastrointestinal digestion conditions. In conclusion, this study demonstrated the feasibility of using tuna bones to prepare a peptides–calcium chelate, which is nutritionally beneficial and has wild application prospects.

## Figures and Tables

**Table 1:** Response surface factors and levels of peptides calcium chelation process.

Levels	Factors			
	A: Peptide/Calcium Mass Ratio	B: pH	C: Time (h)	D: Temperature (°C)
−1	2.5:1	5.0	1.5	60
0	3.0:1	6.0	2	70
1	3.5:1	7.0	2.5	80

**Table 2:** Response surface design and experimental results.

	A: Peptide/Calcium Mass Ratio	B: pH	C: Time (h)	D: Temperature (°C)	Calcium-Chelating Capacity (%)
1	2.50	5.00	2.00	70.00	82.31
2	3.50	5.00	2.00	70.00	84.88
3	2.50	7.00	2.00	70.00	86.35
4	3.50	7.00	2.00	70.00	89.26
5	3.00	6.00	1.50	60.00	90.56
6	3.00	6.00	2.50	60.00	91.78
7	3.00	6.00	1.50	80.00	89.35
8	3.00	6.00	2.50	80.00	90.14
9	2.50	6.00	2.00	60.00	90.59
10	3.50	6.00	2.00	60.00	92.27
11	2.50	6.00	2.00	80.00	89.48
12	3.50	6.00	2.00	80.00	90.09
13	3.00	5.00	1.50	70.00	84.26
14	3.00	7.00	1.50	70.00	88.07
15	3.00	5.00	2.50	70.00	83.19
16	3.00	7.00	2.50	70.00	87.42
17	2.50	6.00	1.50	70.00	90.54
18	3.50	6.00	1.50	70.00	92.43
19	2.50	6.00	2.50	70.00	91.76
20	3.50	6.00	2.50	70.00	93.18
21	3.00	5.00	2.00	60.00	82.93
22	3.00	7.00	2.00	60.00	86.31
23	3.00	5.00	2.00	80.00	80.79
24	3.00	7.00	2.00	80.00	85.22
25	3.00	6.00	2.00	70.00	94.26
26	3.00	6.00	2.00	70.00	94.54
27	3.00	6.00	2.00	70.00	94.01
28	3.00	6.00	2.00	70.00	93.85
29	3.00	6.00	2.00	70.00	93.73

**Table 3:** Analysis of variance of regression equation parameters.

Source	Sum of Squares	df	Mean Square	F Value	*p* Value (Prob > F)	Significant
Models	447.41	14	31.96	103.68	<0.0001	**
A	12.16	1	12.16	39.45	<0.0001	**
B	49.09	1	49.09	159.25	<0.0001	**
C	0.43	1	0.43	1.38	0.2596	
D	5.84	1	5.84	18.94	0.0007	**
AB	0.029	1	0.029	0.094	0.7640	
AC	0.055	1	0.055	0.18	0.6785	
AD	0.001	1	0.001	0.004	0.9506	
BC	0.044	1	0.044	0.14	0.7109	
BD	0.28	1	0.28	0.89	0.3604	
CD	0.046	1	0.046	0.15	0.7044	
A2	4.81	1	4.81	15.62	0.0014	**
B2	365.38	1	365.38	1185.35	<0,0001	**
C2	7.07	1	7.07	22.94	0.0003	**
D2	42.68	1	42.68	138.48	<0.0001	**
Residual	4.32	14	0.31			
Lack of Fit	3.89	10	0.39	3.67	0.1110	Not significant
Pure Error	0.42	4	0.11			
Cor Total	451.73	28				

** Extremely significant (*p* < 0.01).

## Data Availability

The data used to support the findings of this study can be made available by contacting the corresponding author.

## Supplementary Materials

The following supporting information can be downloaded at https://www.mdpi.com/article/10.3390/foods12183403/s1: Figure S1: Chromatograms of TBCP_LMW_ and TBCP_LMW_-ca complex.
