# Supplementary material for: Preparation, Structural Characterization, and Stability of Low-Molecular-Weight Collagen Peptides–Calcium Chelate Derived from Tuna Bones
Source: Foods. 2023 Sep 12;12(18):3403. doi: 10.3390/foods12183403 (PMC10530123; doi:10.3390/foods12183403)
Supplement: Supplementary file 1 [file foods-12-03403-s001.zip › foods-2535024-supplementary.pdf]

Supplementary Material

Figure

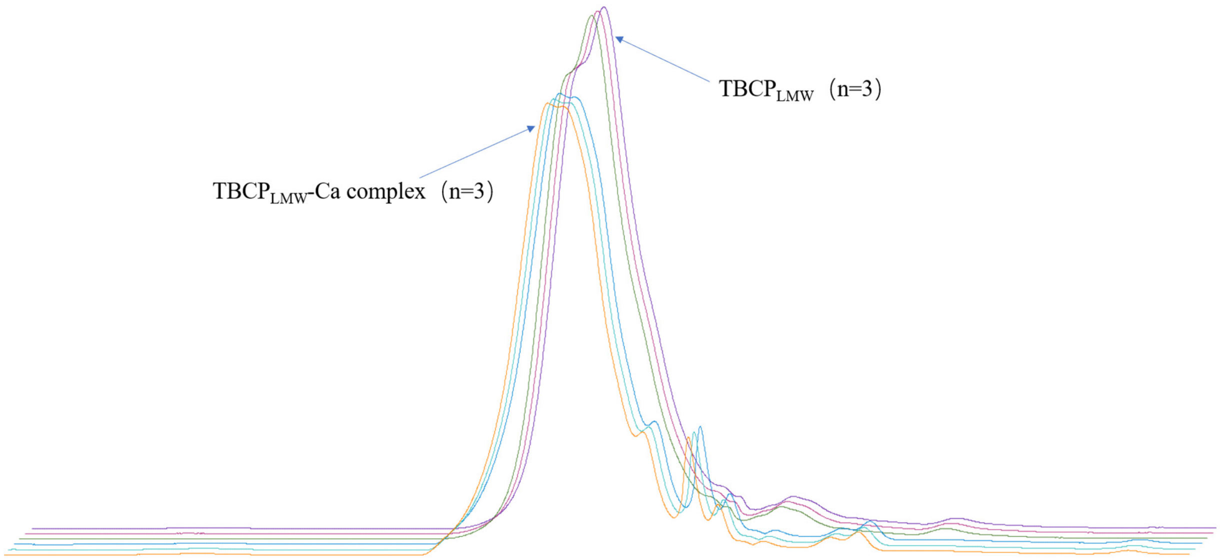

Figure S1. Chromatograms of $TBCP_{LMW}$ and $TBCP_{LMW}$-ca complex.
